# Correction to “Nature-Derived Flame Retardant for Cotton Fabric with Green Coconut Shell”

**DOI:** 10.1021/acs.chas.5c00030

**Published:** 2025-03-07

**Authors:** Tarikul Islam, Abdullah Al Rakib Shikder, Md. Shahabul Hossen, Md Mazbah Uddin, Md Tanvir Hossain, M Mahbubul Bashar

We noticed 2 mistakes in presenting the temperature of our manuscript, which we did not see and correct during proof submission. We apologize for that. We should be more careful in the future.

When we submitted the first revision, it was correct, but we made a mistake (typo mistake) in the second revision submission.

We request that the temperatures 18 °C and 323 °C in our manuscript be corrected/revised to −18 °C and 600 °C in the method section. Changes are highlighted in bold.

In section 2.2.1, the last sentence of the first paragraph should read “The extracted liquid was frozen at **–18** °C for further usage.”

In section 2.2.5, the last sentence should read “The analysis was conducted under a nitrogen atmosphere at a heating rate of 10 °C/min, covering a temperature range up to **600** °C.”

